# Repeated activation of Gαq has a detrimental impact on *C*. *elegans* in an age-dependent manner

**DOI:** 10.1016/j.jbc.2025.110518

**Published:** 2025-07-25

**Authors:** Madison Rennie, Suzanne Scarlata

**Affiliations:** Department of Chemistry and Biochemistry, Worcester Polytechnic Institute, Worcester, Massachusetts, USA

**Keywords:** Phospholipase Cβ, stress granules, *C. elegans*, aging, neuonal signaling, calcium signaling, locomotion

## Abstract

Gαq mediates signals from neurotransmitters to transduce calcium signals through the activation of phospholipase Cβ (PLCβ). In cultured neuronal cells, Gαq stimulation causes two different effects: a calcium-dependent retraction of neurites that requires PLCβ activity and a promotion of stress granules that sequester *ATP5f1b* that is independent of PLCβ activity. Here, we have investigated the effect of single and repeated activation of the Gαq/PLCβ pathway in young (Day 1), middle-aged (Day 4), and old (Day 8) *Caenorhabditis elegans*. While single activation has a slight increase in lifespan, repeated activation is detrimental. Worms that lack PLCβ show high mortality and indicate a need for calcium signaling in early adulthood. Using a combination of fluorescence imaging methods, we followed stress granule formation mediated by activation of Gαq/PLCβ. We find that activation of young worms does not cause the formation of stable stress granules that sequester *ATP51fbl*, but middle-aged worms assemble these stress granules. Disassembly of these particles is slow enough that repeated activation causes their accumulation. Older worms show a severely slowed stress granule dynamic and are largely unresponsive to Gαq/PLCβ activation in terms of stress granule assembly, locomotion, neuronal calcium signaling, and morphological recovery upon stimulation. Taken together, our studies show that PLCβ-mediated mechanisms in young worms are robust but progressively decline with age, which may lead to the accumulation of intracellular aggregates and reduced ability to be stimulated by neurotransmitters and recover after neurotransmitter stimulation.

Organisms are subject to a variety of environmental stressors throughout their lifetime, and the ability to respond to stress depends on the organism’s age and fitness level. *Caenorhabditis elegans (C. elegans)* are an attractive model system to study age-related responses to environmental changes due to their short life span and ease of genetic manipulation (see (1) for review). The short lifespan of *C. elegans* also makes them a good model system to study neurodegenerative diseases, where the underlying biology and late onset in mammalian systems is difficult to study. In humans, many neurodegenerative diseases involve increased expression of proteins that form intracellular aggregates which interfere with neuronal function as seen in loss of cognition and movement (2), such as poly Q gene of Huntington’s disease or the α-synuclein of Parkinson’s disease (see (3)). The relationship between aging and protein aggregation may also be operative in *C. elegans*, where it has been found that reducing the levels of aggregation-prone proteins increases the lifespan of worms (4, 5). Functionally, aging studies of worms show that sensory stimulation seems to remain intact with age, but muscular functions, such as locomotion, decline (6, 7, 8). Genetic factors that contribute to *C. elegans* longevity have been uncovered (9), and recent studies have screened genes involved in *C. elegans* aging. These genes mediate protein homeostasis, energy production, and mitochondria function, as well as other cell functions (see (9, 10, 11)). However, much less is known about their age-related responses to environmental stress on the cellular level.

Recent studies have found that muscarinic agonists, which stimulate the Gαq/phospholipase Cβ (PLCβ) signaling system can reduce age-related decline of function (6, 12, 13). Our lab studies the Gαq/PLCβ pathway in cultured neuronal cells (see (14)). This pathway is activated when neurotransmitters such as acetylcholine, serotonin, bradykinin, endothelin II and histamine bind to their specific G-protein receptor (15, 16, 17). This binding activates Gαq, which then activates PLCβ, setting off a series of reactions that results in an increase in intracellular calcium levels. We have previously found that in model cultured neuronal cells (PC12), Gαq activation causes irreversible retraction of neurites in the first 15 min of G-protein stimulation and causes the cells to return to a stem-like state after 72 h post-stimulation (18). *C. elegans* has a Gαq/PLCβ signaling system analogous to mammalian cells (19, 20, 21), and we have found that activation of Gαq transiently ruptures *C. elegans* neuronal connections and halts locomotion, which then recovers after ∼30 min, presumably due to endogenous growth factors (18).

In cultured cells, we discovered that Gαq activation impacts protein translation and stress granule formation (22, 23). The connection between Gαq and translation lies in the cellular distribution of PLCβ. Besides localizing on the plasma membrane, where it associates with Gαq, PLCβ has a cytosolic population that inhibits C3PO, the promoter of RNA-induced silencing (22, 24), and also binds to stress granule proteins, such as PABPC1 and G3BP1 (25). Activation of Gαq greatly increases its affinity for PLCβ, causing relocation of the cytosolic PLCβ population to the plasma membrane and the release of its binding partners, promoting RNA-induced silencing and stress granule formation due to the release of stress granule proteins.

Stress granules are halted translation complexes that form when cells are subjected to harsh and/or non-physiological environmental conditions, such as nutrient deprivation, oxidative or toxic agents, and temperature variations (24, 26, 27). The formation of stress granules enables cells to shift cellular processes such as protein synthesis to allow them to better accommodate the specific stress. Typically, stress granules are dynamic structures that form and dissipate within 1 to 2 h after a stress event (25), while aberrant less dynamic stress granules are associated with aging (28). Stress granules form under adverse conditions, but we also find that stress granules form when Gαq becomes activated. In PC12 cells, the stress granules that form as a result of Gαq activation sequester two specific mRNAs, the gene that codes for a subunit of ATP synthase (*ATP5f1b*) and one that codes for a protein that mediates synaptic vesicle formation (*Chgb*). The specificity in transcripts sequestered in Gαq-mediated stress granules sharply contrasts the stress granules formed upon heat shock which contain many different and non-specific miRs and mRNAs (22). Because Gαq signaling is a normal physiological event in most species (29, 30, 31), it is possible that stress granules formed upon Gαq activation will accumulate if re-activation occurs before complete disassembly.

Besides impacting stress granules in model-neuronal cells, Gαq stimulation also causes retraction of neurites into the soma through a combination of calcium-induced cytoskeletal changes, receptor aggregation and endocytosis, and changes in membrane surface area due to phosphoinositol 4,5 bisphosphosphate hydrolysis (32). In cultured cells, these changes are irreversible without the addition of differentiation factors, but in *C. elegans*, neuron morphology and locomotion recover after ∼30 min due to intrinsic growth factors (32). Thus, repeated Gαq stimulation may result in reduced locomotion due to reduced synapse integrity.

Here, we characterized the impact of single and repeated stimulation of EGL-30, the *C. elegans* equivalent of Gαq, as a function of age in terms of mechanosensory neuron function using levels of stimulation appropriate for arousal (33). We find that single Gαq stimulation results in age-dependent changes in the size and number of stress granules, ATP5f1b protein levels, locomotion, and neuronal morphology. Importantly, repeated Gαq stimulation results in the accumulation of stress granules, changes in neuron morphology and reduction of lifespan, and that PLCβ (e*gl-8*) is required for longevity. Taken together, our studies show that recovery of single and repeated neurotransmitter stimulation of Gαq/PLCβ1 in *C. elegans* rapidly declines with age.

## Results

### PLCβ positively impacts *C. elegans* lifespan

In cultured mammalian cells, PLCβ relays calcium signals in response to sensory information (16), differentiation through modulating miR and mRNA populations (18, 34, 35), and protecting *ATP5f1b* in stress granules (23). Notably, all these functions are mediated through Gαq activation, which induces calcium signals, reverses PLCβ inhibition of RNA-induced silencing of select transcripts, and allows mRNA transcript protection.

We determined the impact of PLCβ functions on worm lifespan. Although the genes that transcribe Gαq and PLCβ are not associated with *C. elegans’* longevity (see (36)), we have found that the gene that encodes a subunit of ATPsynthase, *ATP5f1b*, is sequestered in stress granules formed in response to Gαq activation, and this sequestration may impact mitochondrial health, which is closely associated with longevity (37, 38, 39). This idea was investigated by following the lifespan of *C. elegans* after the Gαq/PLCβ pathway was activated once or multiple times.

**Figure 1:**
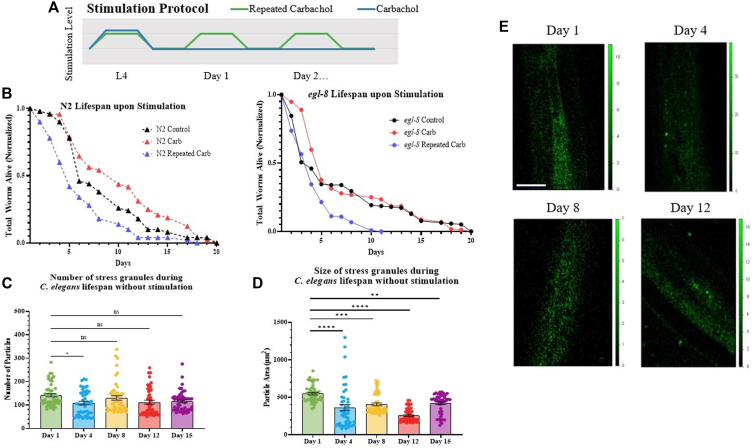
**Survival and accumulation of stress granules during C. elegans lifespan.***A*, cartoon depicting the stimulation protocol used for lifespan studies. L4 larvae were exposed to single Gαq stimulation (1 mM carbachol at room temperature for 30 min) or repeated Gαq stimulation (1 mM carbachol at room temperature for 30 min daily. *B*, survival results for wild-type and *egl-8* mutant worms under control, single, and repeated Gαq stimulation. For all conditions, n = 89 to 103 and three independent repeats were conducted (N = 3). Statistical significance was determined by using simple linear regression in the area of the curve between 80-20%, comparing the carb and repeated carb groups slope values to the control group slope for each strain. For N2, the Carb group (*p* = 0.9850) and Repeated Carb group (*p* = 0.9394) are non-significantly different to the control group although differences at single points are noted in Table S2. For *egl-8*, the Carb group (*p* = 0.3493) is non-significantly different to the control group and the Repeated Carb group (*p* = 0.0016) is significant different to Control. *C* and *D*, age-dependent aggregation of G3BP1::gfp under basal conditions. *C. elegans* head neurons were analyzed at the first day of adulthood up until Day 15. Cumulative values of the number and size of G3BP1::gfp particles were determined by confocal imaging. Data were visualized using GraphPad Prism and analyzed using an ordinary One-Way ANOVA where (∗) represents *p* ≤ 0.1, (∗∗) represents *p* ≤ 0.01, (∗∗∗) represents ≤ 0.001, and (∗∗∗∗) represents *p* ≤ 0.0001. Statistical analysis is conducted by comparing older age groups to Day 1 worms. For all conditions, n = 10 to 15 and two independent repeats were conducted (N = 2). SD values are shown. *E*, representative confocal images of adult worms Day 1 to Day 12 where the y-axis scale represents relative intensity. Scale bar is 20 μm.

Experimentally, worms were synchronized by bleaching (see Experimental procedures) and lifespan recordings began after all worms reached the L4 larval stage. Worms were stimulated by carbachol, a stable form of acetylcholine, either once or repeatedly throughout their lifespan to activate the Gαq/PLCβ1 pathway as shown in the schematic in Figure 1*A*. For single stimulation, worms at the L4 stage were exposed to carbachol for 30 min based on previous studies (32). For repeated stimulation, worms were exposed to carbachol for 30 min at the L4 stage and then everyday thereafter until the end of their life. This acute stimulation protocol was repeated daily with proper amounts of recovery to ensure that *C. elegans* did not enter a Dauer state which would lead to inaccurate lifespan readings.

In N2 wild-type worms, we find that activating Gαq once in young adult worms in the L4 stage before adulthood (*i.e.* Day 1–3) has a positive impact on lifespan seen when worms enter in middle-age (Fig. 1*B*, Table S1), although no significant changes are seen when the single stimulation occurs in middle age or older worms. Alternatively, repeating Gαq activation throughout the worm’s lifetime adversely affects lifespan. These data imply that recovery mechanisms that might have positively impacted lifespan are insufficient during the 23.5 h allotted before the next stimulation. Comparing the curves for untreated, single stimulation, and repeated stimulation, we find that the repeated stimulation preferentially impacts worms in early adulthood as compared to middle-aged and older worms, indicating recovery mechanisms for Gαq stimulation are not well developed early in *C. elegans’* adult life.

PLCβ activity mediates the rise in intracellular calcium upon Gαq activation, whereas mediation of stress granule assembly and RNA-induced silencing does not require active PLCβ. To isolate these functions, we conducted lifespan measurements using a worm strain where PLCβ (*egl-8*) is not expressed (Fig. 1*B*). Comparing the untreated *egl-8* mutants with N2 worms, it is apparent that PLCβ activity is required for longevity. Loss of *egl-8* primarily impacts younger worms as compared to middle-aged and older worms, indicating that PLCβ activity plays an important developmental role early in adulthood. Repeating Gαq activation is especially detrimental to worm longevity, suggesting that both the calcium-dependent and calcium-independent mechanisms contribute positively to lifespan.

### The number of *C. elegans* stress granules in mechanosensory neurons remains constant with age under normal growth conditions

The results above suggest that PLCβ impacts the lifespan of young worms through a mechanism independent of Gαq activation, and through one that requires a time-dependent recovery. Keeping this in mind, we studied stress granules whose assembly is modulated by cytosolic levels of PLCβ that rapidly assemble but have a much slower recovery (23).

Initially, we characterized the size and number of stress granules throughout the lifetime of adult worms that were not subjected to stimulation. For these studies, we quantified the total stress granules in mechanosensory neurons using the stress granule marker G3BP1-eGFP (40, 41, 42), since dimerization of G3BP1 has been shown to be a critical first step in the formation of stress granules (27). Experimentally, these studies were done by collecting confocal images of head neurons in worms expressing G3BP-eGFP (ax2055[gtbp-1::GFP]) and an RFP cytosolic marker for mechanosensory neurons (jsIs973[mec-7p::mRFP]). Using both markers allows us to observe stress granules in mechanosensory neurons by monitoring changes in the green channel while simultaneously selecting neuronal head regions in the red channel.

We analyzed the size and number of stress granules in mechanosensory neurons by collecting confocal images and creating a series of optical slices spanning the width of the whole organism, identifying particles ∼10 μm^2^ or greater. The results are shown in Figure 1, *C* and *D*. We find that in adult worms aged 1 to 15 days, the average number of stress granules in neurons remains constant, although the average size of the stress granules decreases with age. Thus, aging is associated with reduced size of stress granules without a change in number.

We connected the presence of stress granules with lifespan by subjecting young worms (*i.e.* Day 1, 2, and 3) to the same repeated Gαq stimulation protocol described in Figure 1*A* (*i.e.* This age range occurs prior to middle-age, where Gαq-mediated egg-laying occurs (43) and prior to older ages, which are not well stimulated by carbachol (*see below*). The results (Fig. S1), show that Gαq stimulation does not lead to significant stress granule accumulation in the early stages of adulthood. This result, coupled with nerve morphology studies as detailed below, indicates that reduced lifespan with daily repeated activity occurs in processes during middle age.

### Repeated Gαq stimulation increases the number and size of Ago2-related stress granules

**Figure 2:**
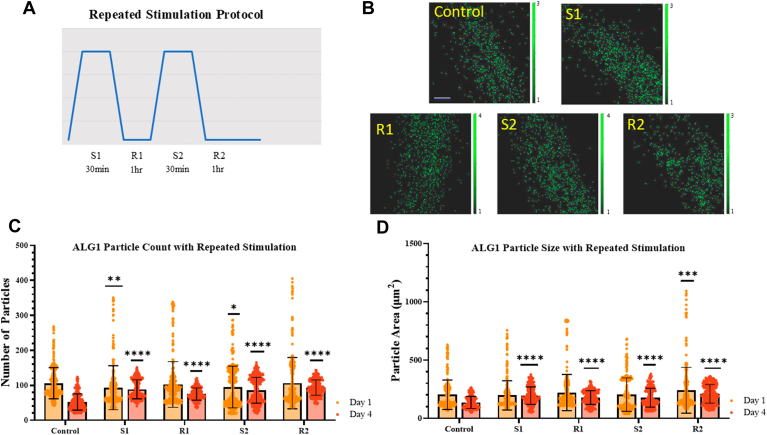
**Ago2-associated stress granules with repeated Gαq activation and recovery.***A*, cartoon depicting the repeated stimulation protocol used for studies where worms were stimulated for 30 min and allow to recover for 60 min. *B*, representative confocal images of Day 1 adult worms under control and repeated stimulation conditions where the y-axis scale represents relative intensity. Scale bar is 20 μm. *C* and *D*, aggregation of ALG-1 in Day 1 and Day 4 adult worms head neurons. Confocal images taken under control conditions, immediately following stimulation of Gαq (S1), and immediately following recovery (R1). This cycle of stimulation and recovery was repeated a second time as well (S2 + R2). Data were visualized using GraphPad Prism and analyzed using a Two-Way ANOVA, where “ns” correlates to non-significance, (∗) represents *p* ≤ 0.1, (∗∗) represents *p* ≤ 0.01, (∗∗∗) represents *p* ≤ 0.001and (∗∗∗∗) represents *p* ≤ 0.0001. Statistical analysis is conducted by comparing treated groups to control conditions in each age worm. For all conditions, n = 10 to 15 and three independent repeats were conducted (N = 3).

Ago2 is the key nuclease component of the RNA-induced silencing complex and will form stalled complexes (*i.e.* stress granules) when there is imperfect pairing between the Ago2-bound miR and mRNA (44, 45). Ago2 stress granules appear to be a subset of G3BP1-containing stress granules, as indicated in previous studies (23) and are much smaller in size as compared to G3BP1 (compare Figs 1*D*–2*D*). As noted, we have found that Ago2 particles have a distinct protein and RNA content (22) and, important for this study, the formation of these particles is dependent on Gαq activation and cytosolic PLCβ levels.

Our results in Figure S1 indicate that stress granules in Day 1 to 3 worms recover when stimulated daily. Based on our previous results in PC12 cells, suggesting that stress granules disassemble ∼30 min after stimulation and that worm locomotion recovers after 30 min (23, 32), we wanted to test whether a more rapid stimulation/recovery protocol would lead to stress granule accumulation. To this end, we followed changes in the size and number of stress granules containing ALG-1, the Ago2 equivalent in *C. elegans* (46, 47), in Day 1 adult worms with single and repeated Gαq activation, where the recovery time was only 1 h (Fig. 2, *A*). Day 1 worms showed little significant change in either the number or size of Ago2-particles (Fig. 2, *C* and *D*), although the distributions of these parameters are very broad. This result indicates that either the assembly of Ago2 stress granules with Gαq stimulation is low or that disassembly of these particles occurs within the recovery period.

We compared the results of young worms with Day 4 worms. In contrast to Day 1, the number of particles in Day 4 worms increased with stimulation and did not recover to basal levels during the time of the experiment (Fig. 2, *C* and *D*). Overall, these studies suggest that stress granules associated with Ago2 accumulate with repeated Gαq activation.

### Gαq stimulation changes the level of ATP5f1 in an age-dependent manner

**Figure 3:**
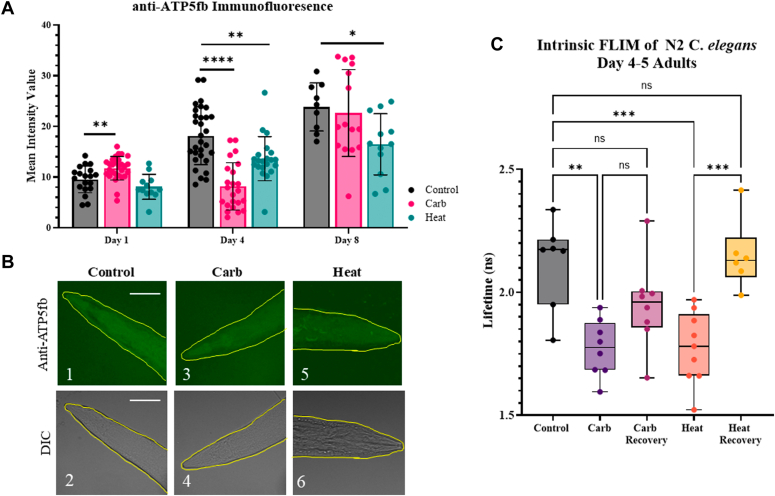
**Changes in ATP5f1 levels and redox state with Gαq stimulation and heat shock.***A*, ATP5f1 immunofluorescence intensity upon different stress conditions. Wildtype worms were collected at adult Day 1, 4, and 8 stage and analyzed for ATP5f1 levels by immunostaining with a monoclonal antibody under control, Gαq stimulation by treating with 1 mM carbachol at room temperature for 30 min, and heat shock at 35 °C for 15 min. All intensity values were background subtracted and normalized to an unstained control. Data were visualized using GraphPad Prism and analyzed using student t-tests comparing two groups where (∗) represents *p* ≤ 0.1, (∗∗) represents *p* ≤ 0.01, and (∗∗∗∗) represents *p* ≤ 0.0001. Statistical analysis was conducted by comparing treated groups to control conditions in each age worm. For all conditions, n = 9 to 29 and four independent repeats were conducted (N = 4). *B*, representative confocal images of Day 1 adults under control, carbachol, and heat stimulation. Scale bars are 10 μm. ROI regions used to analyze the fluorescent values are in *yellow*. The ROI regions were first determined by the DIC image (1, 3, and 5) and then placed on the fluorescent image (2, 4 and 6) for analysis. *C*, changes in lifetime of the intrinsic fluorescence of Day 4 to 5 worms, which is related to the redox state. Worms were analyzed under control, Gαq stimulation by treating with 1 mM carbachol at room temperature for 30 min, and heat shock at 35 °C for 15 min conditions where stimulation periods were followed by a 30 min recovery. Worms head regions were imaged. Data were visualized using GraphPad Prism and analyzed using an ordinary One-Way Anova, where “ns” correlates to non-significance, (∗∗) represents *p* ≤ 0.01, (∗∗∗) represents *p* ≤ 0.001, and (∗∗∗∗) represents *p* ≤ 0.0001. Statistical analysis was conducted by comparing treated groups to control conditions and additionally by comparing treatment groups to recovery groups. For all conditions, n = 6 to 9 and three independent repeats were conducted (N = 3).

In PC12 cells, activation of Gαq sequesters the mRNA, *ATP5f1b,* in stress granules to protect the cell’s energy sources, and this effect is seen by a transient decrease in *ATP5f1b* levels with Gαq activation (22). Specific sequestration of transcripts is not seen in stress granules formed upon heat shock. To determine whether this is also the case for stress granules formed around mechanosensory neurons in *C. elegans*, we measured changes in the amount of the ATP5f1 protein under control, Gαq activation, and heat stress in Day 1, 4 and 8 worms noting that attempts to measure changes in mRNA level were unsuccessful due to the difficulty of preparing samples from the whole organism. These studies were done by quantifying and comparing changes in immunofluorescence intensity using a monoclonal ATP5f1 antibody. Representative confocal images of Day 4 worms are shown in Figure 3*B*.

Day 1 worms show a slight increase in ATP5f1 levels with Gαq stimulation and a decrease with heat (Fig. 3*A*). This behavior correlates with the inability of these worms to assemble stress granules and argues against their formation and rapid disassembly. In contrast, Day 4 worms showed a clear reduction in ATP5f1 levels, supporting the sequestration of *ATP5f1b* in Ago2 stress granules (Fig. 3*A*). Heat shock showed a smaller reduction of ATP5f1 levels correlating with a reduction of protein production and non-specific sequestration of RNAs in stress granules (22). Day 8 worms, which displayed higher ATP5f1 levels despite the observation that they have diminished ATP stores and mitochondrial function (48, 49) (see Discussion), showed little change with Gαq stimulation and a small decrease with heat shock (Fig. 3*A*).

The studies above were supported by assessments of the redox state of the whole organism, which is related to ATP levels, by monitoring changes in intrinsic fluorescence. In these measurements, the lifetimes associated primarily with the intrinsic fluorescence of NAD(P)+/NAD(P)H report on the oxidation state (50, 51). Using Day 4 to 5 worms, we find that Gαq stimulation reduces the overall lifetime, corresponding to increased oxidation (Fig. 3*C*), which may begin to recover after the stimulus is withdrawn. In contrast, worms subjected to heat shock show increased oxidation that fully recovers. The observation that Gαq stimulation impacts redox through ATP levels whose average intrinsic lifetime values remain lower than that of control conditions is consistent with sequestration of *ATP5f1* into stress granules that do not fully disassemble. Heat shock, which non-specifically sequesters RNAs and broadly affects that RNAs of redox-associated proteins, recovers more rapidly.

### Aging impacts the assembly and disassembly of Gαq-mediated stress granules

Because Ago2 is a subset of stress granules, we more fully characterized the canonical stress granule marker G3BP1 in *C. elegans* at different ages. These studies entailed using a fluorescent tagged marker, G3BP1-eGFP (40) focused on mechanosensory neural regions. In an initial approach, we followed the onset of G3BP1-eGFP oligomerization, the hallmark of stress granule formation, by number and brightness (N&B) analysis. In this method, a time series of images are collected and the movement of each pixel in the image is followed. By analyzing the movement of pixels with time, and comparing these to control samples, we can determine the relative number of fluorophores in a diffusing species and their associated intensities through N&B analysis (52) (see Experimental procedures).

**Figure 4:**
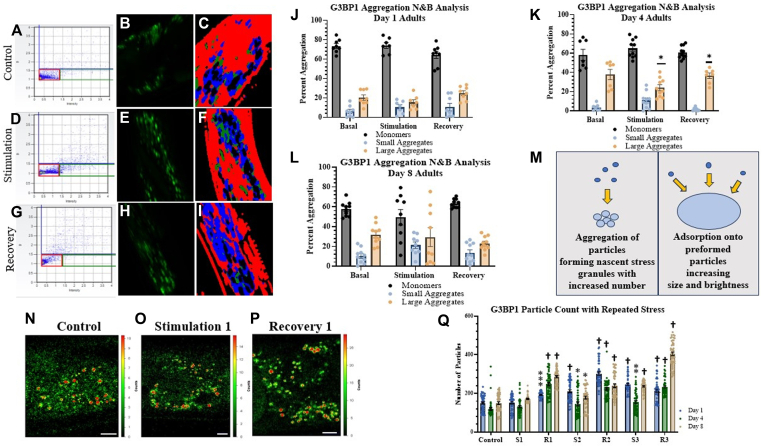
**Age-dependent changes of G3BP1 stress granule assembly upon repeated Gαq stimulation and recovery.***A*–*I*, representative graphs and images of Day 4 *C. elegans* N&B results. Graphs in the first column (*A*, *D*, *G*) display N&B values for each pixel where the y-axis corresponds to the brightness of the particle and the x-axis corresponds to particle intensity. The pixels contained in the *red boxes* are the values found for free, non-aggregated eGFP. Points outside the *red box*, shown in *green* and *blue boxes*, correspond to higher-order species of G3BP1 where the percent of pixels in these categories are summed and given in Table S2. Images in the second column (*B*, *E*, *H*) correspond to the fluorescence microscopy images. The third column of images (*C*, *F*, *I*) highlight the pixels in the fluorescent microscopy image correlated with the pixels indicated in the (*B*) vs. (*I*) graphs. Pixels are color coded based off level of aggregation, the *red* pixels correlating to the control monomers in *red box*, and the *green* and *blue* pixels correlating to the small and large aggregates. Therefore, the values graphed in the *red box* (*A*, *D*, *G*) are highlighted in *red* color in images (*C*, *F*, *I*); and the same is true for the *green* and *blue box*. Worms were treated with 1 mM carbachol for 30 min to stimulate Gαq and allowed to recover on OP50 plates for 1 h. *J*–*L*, aggregation values upon Gaq stimulation for Day 1, Day 4, and Day 8 worms. Cumulative values for monomeric protein and small and large aggregates are based off the brightness and intensity values recorded. Data were visualized using GraphPad Prism and analyzed using a Two-Way ANOVA where (∗) represents *p* ≤ 0.1. Statistical analysis is conducted by comparing small and large aggregate groups to monomeric conditions in each treatment group. For all conditions, n = 7 to 13 and three independent repeats were conducted (N = 3). SD values are shown. *M*, model highlighting major mechanisms of stress granule formation captured by N&B analyses. *N–P*, representative confocal images of Day 1 adult worms upon control, stimulation, and recovery conditions. Scale bars are 10 μm. Colored bars to the *right* of each image describe the scale of photon counts for each condition. *Q*, number of G3BP1 stress granules in *C. elegans* head neurons at Day 1, Day 4, and Day 8. Aggregation was observed using confocal imaging under control conditions, immediately following 1 mM carbachol exposure to stimulate Gαq (S1) for 30 min at room temperature, and after recovery (R1) where carbachol exposure was removed and the worms were allowed to recover on a seeded NGM plate. This process was repeated for a second (S2 and R2) and third cycle (S3 and R3). Data were visualized using GraphPad Prism and analyzed using a Two-Way Anova, where “ns” correlates to non-significance, (∗) represents *p* ≤ 0.1, (∗∗) represents *p* ≤ 0.01, (∗∗∗) represents *p* ≤ 0.001, and (†) represents *p* ≤ 0.0001. Statistical analysis is conducted by comparing treated groups to control conditions in each age worm. For all conditions, n = 10 to 15 and three independent repeats were conducted (N = 3). SD values are shown.

Briefly, when the fluorescent-tagged G3BP1 oligomerizes upon stress granule formation, the brightness of the diffusing species increases due to the increased number of fluorophores in the particle and increased particle size. We can plot the brightness and intensity (B & I) values of each pixel in the image and select the area of the plot that corresponds to control conditions before stimulation, as shown in Figure 4, *A*–*I* and described below. Increased aggregation is indicated if the B&I points of the sample lie outside the pixels that correspond to control conditions (*i.e.,* the diffusing particle contains either more fluorophores or is of higher intensity). There are two types of outlier points displayed in Figure 4, *A*–*I*, those that correspond to larger complexes without increases in brightness (referred to as *small aggregates*) and those that are higher-order oligomers (referred to as *large aggregates*). It is important to note that this method is only sensitive to diffusing species, and large aggregates that do not diffuse during the time sequence will not be observed. Thus, this method will be sensitive to the formation of nascent particles containing G3BP1-eGFP.

We used N&B to follow the assembly of stress granules immediately after stimulation in real time, and representative images are shown in Figure 4, *A*–*I* of Day 4 worms subjected to Gαq stimulation and recovery. The compiled results for Day 1, 4, and 8 worms are shown in Figure 4, *J*–*L* with cycles of 30-min activation of Gαq by carbachol addition and 30-min recovery periods by removal of stimulant. On Day 1 and on Day 8, worms (Fig. 4, *J* and *L*), we could not detect changes in aggregation. However, Day 4 worms showed significant differences (Fig. 4*K*).

Before interpreting these data, we note that changes in N&B may reflect two major mechanisms; 1-the formation and dissolution of nascent G3BP1 particles resulting in shifts to higher N&B values; 2- G3BP1 and/or associated proteins absorb onto slow-moving pre-formed particles causing particles to become undetectable by N&B and resulting in little changes in the N&B plots (see Fig. 4*M*). Thus, based on our Ago2 results and the confocal results presented below, we believe that Day 1 worms do not form stable nascent particles while Day 4 worms dynamically assemble and disassemble particles through the two major mechanisms shown in Figure 4*M*. Values for Day 8 change little with stimulation or recovery. These results indicate that the formation of nascent stress granules with carbachol stimulation is age-dependent, with middle-aged worms showing an increase in particles, while young and older worms show little net change.

Because N&B can only capture the formation and dissolution of small particles, confocal imaging was used to observe larger G3BP1-eGFP particles in mechanosensory neurons during repeated Gαq/PLCβ activation, and some representative images are shown in Figure 4, *N*–P. Monitoring the number of particles with repeated stimulation/recovery cycles indicates that the initial cycles of stimulation do not impact the number of particles, but rather assembly of G3BP1 particles occurs in the recovery phase. Moreso, an increasing trend with stimulation and recovery is seen in all age groups (Fig. 4*Q*). These data suggest that repeated Gαq activation and recovery promote the number of G3BP1 particles as *C. elegans* aging occurs.

We compared the formation of G3BP1 stress granules produced by Gαq activation in adult Day 4 *C. elegans* to those formed upon heat stress, whose ability to generate stress granules has been well described (53, 54) (Fig. S2). To prevent pervasive heat damage (see (55)), we shortened the stress times from 30 to 15 min and increased the recovery time to 1 h. We find that heat shock results in a steady increase in particle number that continues to rise during recovery, and these changes are accompanied by a small increase in particle size. These results show that, in contrast to single Gαq activation, subjecting worms to heat shock results in systematic and irreversible increases in the overall number of G3BP1-associated stress granules.

### Aging impacts the time dependence of Gαq stimulation changes in locomotion

In previous studies, we found that Gαq stimulation causes contraction of *C. elegans* neurons that disrupts neural connections and locomotion (32). In cultured cells, retraction can only be reversed by the addition of nerve growth factor, but for Day 5 *C*. *elegans*, reversal occurs naturally after the stimulus is removed. Because Gαq stimulation disrupts neuronal connections (32), we studied the effect of Gαq stimulation on movement by assessing *C. elegans* locomotion.

**Figure 5:**
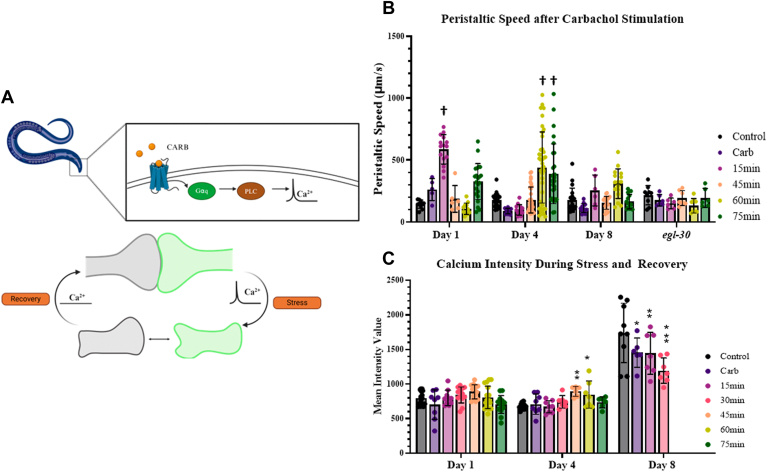
**Recovery of C. elegans’ peristaltic speed and calcium signaling after Gαq stimulation.***A*, model of neuronal disruption upon stimulation and recovery. Figure made with BioRender. *B*, peristaltic speed of adult Day 1, 4 and 8 wildtype *C. elegans* and in Day 1 *egl-30* mutants during control, Gαq stimulation and recovery conditions. Worms were stimulated with 1 mM carbachol at room temperature to stimulate Gαq and then seeded on a NGM OP50 plate at room temperature for 15-80 min during recovery. Peristaltic speed ([forward track length + reverse track length]/time) was recorded using an inverted camera and WormLab software from MBFBioscience. Videos were recorded for 1 min and analyzed using WormLab camera software. Data were visualized using GraphPad Prism and analyzed using an ordinary One-Way Anova, where (†) represents *p* ≤ 0.0001. Statistical analysis is conducted by comparing treated groups and recovery groups to control conditions in each age worm. For independent conditions, n-values are reported as follows - Day 1 wildtype (n = 6–22), Day 4 wildtype (n = 6–20), Day 8 wild-type (n = 12–49), and *egl-30* (n = 6–9). Two independent repeats were conducted (N = 2). SD values are shown. *C*, calcium levels of adults Day 1, 4 and 8 *C. elegans* during control, Gαq stimulation and recovery conditions. Calcium levels were recorded utilizing a GcAMP tag. Worms were stimulated with 1 mM carbachol at room temperature to stimulate Gαq and then seeded on a NGM OP50 plate at room temperature for 15-80 min during recovery. For day 8 worms, worms only survived 45 min, and after 45 min no live worms were detected. Data were visualized using GraphPad Prism and analyzed using an ordinary One-Way Anova, where (∗) represents *p* ≤ 0.1, (∗∗) represents *p* ≤ 0.01, (∗∗∗) represents *p* ≤ 0.001. Statistical analysis is conducted by comparing treated groups and recovery groups to control conditions in each age worm. For all conditions, n = 6 to 17 and two independent repeats were conducted (N = 2). SD values are shown.

Changes in *C. elegans* peristaltic speed (56) after carbachol addition at various time points (Fig. 5*B*) were measured in N2 Day 1, 4, and 8 worms using the same G3BP1-eGFP tagged worm as described above. Peristaltic speed takes into account the forward and reverse distances travelled when calculating the worm's speed over time (see Experimental procedures). We find that Day 1 worms show an initial increase from 180 to 200 μm/s after the addition of carbachol that reaches a maximum average of 600 μm/s after 15-min post-Gαq stimulation. In contrast, Day 4 worms showed an initial drop in speed in the first 30 min post-stimulation and an average maximum of ∼500 μm/s, which is not reached until 60 min post-stimulation. Like Day 1 worms, the speed of Day 4 worms recovered after their maximum speed was obtained. Day 8 worms showed little response to stimulation, and their speeds were much slower (200–360 μm/s) in accord with previous studies showing functional decline in motor activity (57) in older worms. These results highlight the strong age-dependence in the maximum peristaltic speeds initiated by Gαq stimulation.

To show that locomotion changes are dependent on the activation of the *egl-8/egl-30* cascade, egl*-30* mutant worms were subjected to carbachol stimulation and measured for peristaltic speed as described above. In Day 1 adults, the *egl-30* mutants show a lack of response to carbachol stimulation, and worms show stable locomotory patterns throughout the recovery time (Fig. 5*B*). These studies confirm that EGL-8 mediates changes in locomotion due to carbachol activation.

The lag in increased peristaltic speed and the time of recovery is not likely be related to stress granules based on the short time scale. Rather, their differences could be related to the calcium-dependent morphology changes induced by activation of the GPCR/PLCβ1 signaling system (32), or analogous pathways that mediate acetylcholine release from the ventral cord motor neurons in chemosensation (58). To better understand movement, we assessed calcium responses during stimulation and recovery using a GcAMP reporter (59) on Day 1, 4, and 8 worms (Fig. 5*C*). In Day 1 adults, the calcium levels have little change upon GPCR stimulation and then slowly increase after ∼15 min of recovery. Day 4 worms show increased levels of calcium 30-45 min post stimulation, which is around the same time we see locomotion recovery (Fig. 5*B*). Day 8 worms show increased levels of calcium production in comparison to Day 1 and 4, and these calcium signals drop in intensity after stimulation and during recovery. Taken together, these data suggest a strong and expected link between calcium recovery after GPCR stimulation and intracellular calcium return to allow basal neuronal connectivity, see Figure 5*A*.

### Repeated Gαq stimulation alters neuron morphology

**Figure 6:**
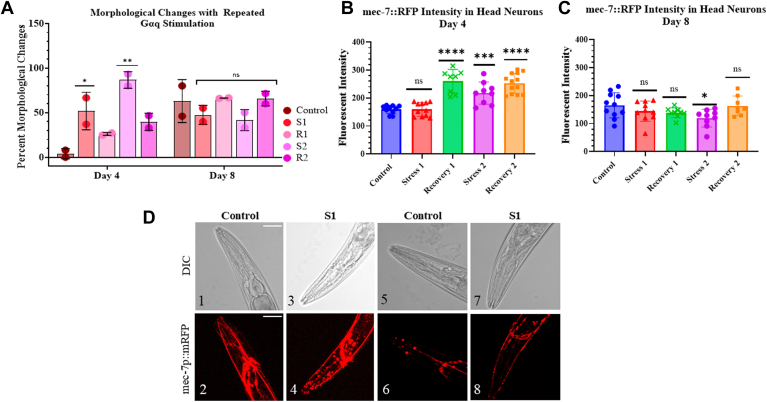
**Percent of abnormal morphology observed in mechanosensory neurons upon repeated Gαq stimulation and recovery.***A*, total morphological changes were compiled for worms under control, one round of Gαq stimulation by 1 mM carbachol at room temperature (S1), and 1 h recovery at room temperature on an OP50 seeded NGM plate (R1), followed by a second cycle of stimulation and recovery (S2 and R2). To visualize morphology throughout the worms’ head region, z-stack imaging was utilized which captured 1um thick slices throughout the worms’ head. Data were visualized using GraphPad Prism. Data were visualized using GraphPad Prism and analyzed using a Two-Way Anova and Šídák's multiple comparisons test, where “ns” correlates to non-significance, (∗) represents *p* ≤ 0.1, and (∗∗) represents *p* ≤ 0.01. Statistical analysis is conducted by comparing treated groups and recovery groups to control conditions in each age worm. For all conditions, n = 6 to 17 and two independent repeats were conducted (N = 2). SD values are shown. *B-C*, fluorescent measurements of beta-tubulin in Day 4 and Day 8 head neurons. *C. elegans* tagged with mec-7::RFP were collected and assayed for neuron integrity using the same stimulation and recovery protocol. Data were visualized using GraphPad Prism and analyzed using unpaired t-tests where “ns” correlates to non-significance, (∗) represents *p* ≤ 0.1, (∗∗∗) represents *p* ≤ 0.001, and (∗∗∗∗) represents *p* ≤ 0.0001. Statistical analysis is conducted by comparing treated groups and recovery groups to control conditions in each age worm. For all conditions, n = 8 to 13 and SD values are shown. *D*, representative confocal images of Day 4 worms (1–4) and Day 8 worms (5–8) under control and stimulation (S1) conditions. Scale bar is 20 μm.

Because the onset of increased peristaltic speed may be due to the ability of the synapses to recover after Gαq stimulation (32) (Fig. 5*A*), we measured the changes in the morphology of *C. elegans* mechanosensory neurons in the head, focusing on the nerve ring and dendritic spine structures with single and repeated Gαq activation (Fig. 6). In Figure 6*A*, we plot the total number of observed morphological changes (*i.e.* beading and altered morphology), as seen using a 63x objective (Fig. 6*D*). Under control conditions, neurons in Day 4 worms do not show abnormal morphology but show extensive changes with stimulation that reverse with recovery. This same behavior is seen during the second cycle of stimulation and recovery and is consistent with the initial reduction in locomotion (Fig. 5*B*). Alternately, Day 8 worms show a significant number of morphological abnormalities under control conditions that decrease with Gαq stimulation, and this trend reverses during recovery, with similar trends in the second application of stimulation and recovery. These abnormal morphologies seen with Gαq stimulation will alter the contraction of the underlying muscle and may be related to the observed changes in locomotion. Recent work by Tang-Schomer and colleagues (60) observed that beads formed along axons can be observed as a protective response and can be induced by calcium elevation.

In a second round of studies, *C. elegans* tagged with mec-7::RFP were collected and assayed for neuron integrity (Fig. 6, *B* and *C*). The marker mec-7 indicates beta-tubulin structures in the mechanosensory neurons found in the head, and when attached to a fluorescent protein, provides a direct readout of neuron integrity. In Day 4 worms, we see significantly increased beta-tubulin intensity after the first stimulation, which remains elevated even after another cycle of stimulation. In Day 8 worms, however, no significant differences are reported at any condition consistent with the trend in Figure 6*A*.

## Discussion

**Figure 7:**
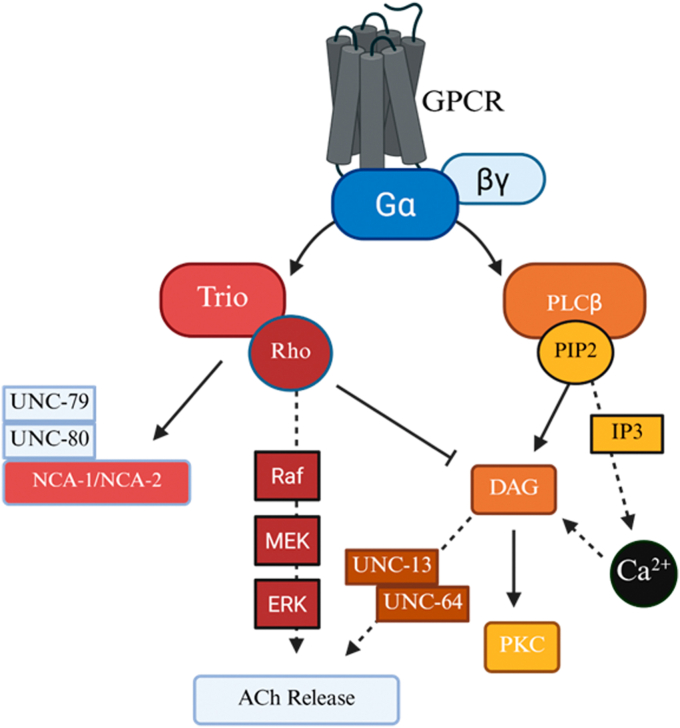
**Potential role Gαq signaling pathways in *C. elegans* aging.** Depicted are the two major downstream branches of Gαq signaling initiated by GPCR activation, where the *left* shows Gαq activating RhoGEF Trio, that activates Rho, and Rho then activates the MAPK cascade (Raf → MEK → ERK), which regulates gene expression and stress responses (see (83)) and modulates auxiliary subunits UNC-79/UNC-80, of NCA-1/NCA-2 sodium leak channels, influencing neuronal excitability and acetylcholine (ACh) release (see (84)). The *right* shows how Gαq activates PLCβ, which hydrolyzes PIP_2_ into IP_3_ and DAG to mobilize intracellular Ca^2+^, while DAG activates PKC, which modulates UNC-13 and UNC-64 (85) that are essential for synaptic vesicle priming and fusion. As detailed in the text, these pathways collectively regulate processes that are tightly linked to *C. elegans* aging and lifespan regulation.

In this study, we characterized the role of PLCβ in mediating calcium-dependent and calcium-independent responses in different age groups of *C*. *elegans*. As shown in Figure 7, in *C. elegans*, PLCβ and Gαq mediate proteins involved in stress and neural transmission, which both change during aging. Here, we find that PLCβ plays a role in defining longevity as seen by the positive impact of single Gαq activation lifespan, the negative impact of repeated activation, as well as the profound detrimental effects of ablating PLCβ on lifespan. Stimulation of Gαq (EGL-30), whose gene has ∼80% homology to mammalian Gαq (21), is a normal physiological event that is positively associated to memory function through calcium-mediated activation of CREB (61), and is associated with *C. elegans* locomotion (see (62)). Recent studies (63) found that expressing constitutively active Gαq (EGL-30) preserves long-term associative memory in older worms and Gαq activation slows memory decline (64). Note that this Gαq mutation does not impact locomotion, suggesting that its effect on worm health is not simply due to elevated basal calcium levels, and that other mechanisms may be involved. In contrast to constitutively active EGL-30, *C. elegans* with other EGL-30 mutations have egg-laying defects, reduced viability, pharyngeal pumping, and movement (see (65)). Together, continuous Gαq activity as seen in those mutants has positive effects, presumably due to adaptive mechanisms, whereas defects in this pathway are detrimental, and these effects may be calcium independent.

Our studies find that single Gαq stimulation has a small positive effect on wild-type (N2) *C. elegans* lifespan that is observed in middle-aged and older worms, while repeated stimulation is detrimental. Studies using mutant worms where EGL-8 is not produced suggest that this positive impact of single stimulation occurs through calcium-dependent mechanisms in early adulthood showing the need for calcium signals during maturation. The reduced lifespan observed in these germline-containing worms are consistent with reduced viability of Gαq (*egl-30*) mutants but contrasts the positive impact of *egl-8* mutants in germline-deficient worms (see (66)). This former comparison may be traced to the ability of PLCβ to promote differentiation and halt proliferation through modulation of miR populations (18) in cultured cells while germline-deficient worms would be expected to have different miR populations and shifted protein populations that could impact lifespan. Specifically, we believe that adaptation to constitutively active protein includes prolonged elevated calcium that may aid in memory formation but would inhibit the dynamic aspects of cytosolic PLCβ functions as well as the onset and extent of neuronal retraction.

Depending on environmental conditions, *C. elegans* hermaphrodites usually begin laying eggs close to or after Day 4 and stop around Day 10 (67), and Gαq plays a key role in egg-laying through its ability to contract muscles (43). Our data show that young worms fully recover from Gαq stimulation and older worms lack the full ability to be stimulated. These observations lead to the speculation that repeated Gαq stimulation primarily impacts middle-aged worms and that this impact may be through egg-laying, possibly through enhancing the muscle contractions required for this process. If *C. elegans* strive to lay as many eggs as possible during their lifetime and Gαq stimulation enhances egg-laying, then this would be consistent with a reduction in lifespan. Support for this idea comes from studies showing that worms maximize their number of progenies to stabilize their population so that if their lifespan is prolonged, their fertility declines (68). Research probing the complex connections between Gαq, age and egg-laying would bear this out.

To understand the effects of single and repeated Gαq stimulation on the neuronal health of *C. elegans,* we first examined the impact of Gαq-mediated stress granule formation in the context of aging (23). Cellular aging is accompanied by loss in mitochondria function and protein production, both of which impact Gαq activation through loss in coupled receptors which diminish responses and through the sequestration of *ATP5f1b* mRNA in Gαq-specific stress granules. Stress granules can be formed by different environmental perturbations and those assembled by Gαq activation are expected to have different compositions and properties than those formed under other treatments. For example, Ching and coworkers found that dietary restriction in *C. elegans* mediates stress granule formation through a pathway distinct from heat shock (69). We reasoned that repeated Gαq may impact worm lifespan, in part, through the formation of stress granules. This idea is based on cell culture studies (23) and *C. elegans* studies showing that intracellular aggregates, which may include the assembly of stable stress granules, are found in many neurodegenerative diseases (70, 71, 72).

Previously, David and coworkers followed protein aggregates in *C. elegans* as a function of age by characterizing the amount and content of insoluble protein aggregates (5). Here, we followed changes in G3BP1-associated stress granules using the canonical stress granule marker G3BP1 in adult *C. elegans* from Day 1 to 15 (Fig. 1, *C* and *D*). We delineated changes in stress granules in terms of number which corresponds to assembly and disassembly of stress granules, and size which corresponds to the association or dissociation of smaller G3BP1 to larger complexes, such as stalled ribosomes. In contrast to observing an increase in protein aggregation with age, we find that the number of small G3BP1 stress granules remain fairly constant although there is a decrease somewhat in size as seen by confocal imaging. These results suggest that the larger aggregates seen in previous studies (5) may correspond to other complexes formed from dysfunction in protein degradation, or irreversible entanglement in disordered regions of proteins that result from reduced proteosome activity rather than new stress granule protein-associated aggregations forming (73).

While the impetus for studying the assembly and disassembly of stress granules came from our previous studies in cultured cells, it is important to stress the experimental differences in monitoring stress granules in *C. elegans versus* cultured cells. In cultured cells, we can follow changes in stress granules directly in real time and we find stress granule assembly occurs within ∼2 min where it then recovers over ∼20 to 30 min (23), while here, worms were continuously treated with stimulant for 30 min before removing and viewing, then allowing recovery to occur. While this experimental protocol does not allow us to view initial increases in stress granule number and size, it does allow us to compare changes in stress granules with age and with repeated stimulation. We note that compared to undifferentiated PC12 cells, *C. elegans* mechanoneurons contain ∼10 fold less particles that are ∼10 fold larger in size under basal conditions. One possible reason for this difference is that stress granules in undifferentiated cells contain a higher percentage of numerous miRs, while stress granules in differentiated cells contain higher levels of mRNA.

The results for Ago2 stress granule formation as well as ATP5f1 levels suggest that Day 1 worms assemble and disassemble Ago2 stress granules rapidly and do not protect *ATP5f1* as compared to older worms. Although Day 1 worms show fast recovery to Ago2 stress granules formed in response to Gαq activation, we find that they will accumulate G3BP1 stress granules, which are larger and include all types of stress granules. Worms in all age groups showed incomplete recovery of G3BP1 aggregation after Gαq stimulation, suggesting that stress granules may accumulate with repeated stimulation, and the propensity for accumulation appears to increase in older worms (Fig. 4*Q*). It is tempting to speculate that these accumulated complexes underlie neurodegenerative disorders (see (74)). Comparing stress granule behavior in response to repeated Gαq stimulation with heat shock shows that both conditions result in stress granule accumulation (Fig. S1). We note that the RNA content of the accumulated stress granules formed in response to heat complexes is less specific compared to the more regulated Gαq-induced stress granules as is the case for PC12 cells (22) (Fig. 3*A*).

In cultured cells, Gαq activation promotes the formation of Ago2 stress granules that protect and sequester two RNAs, *Chgb* and *ATP5f1b* (see (22)). We tested whether this is the case in *C. elegans* neurons. Monitoring ATP5f1 levels by immunofluorescence in Day 1, 4, and 8 worms show age-dependent changes that mirror their respective stress granule assembly (Figs. 3*A* and 4*Q*). We find that maximal protection of ATP5f1, as seen by reduced protein production, is highest for Day 4 worms, most likely due to rapid recovery of Day 1 worms and the reduced stress granule formation of Day 8 worms. We note that immunostaining of ATP5f1 in Day 8 worms was more intense than Day 1 and 4. We believe these higher intensities are due to better accessibility of the antibody to the epitope due to loss of mitochondrial integrity rather than higher expression levels in Day 8 worms (37, 49). Efforts to determine levels by western blotting were unsuccessful.

In addition to immunostaining, we assessed the protection of ATP5f1 with Gαq stimulation, by monitoring changes in the redox state of cells to assess mitochondria health and subsequently ATP production by measuring the changes in intrinsic fluorescence contributed by NAD(P)H, where these values reflect the optical redox state of mitochondria (50). Upon Gαq stimulation we see a significant decrease in lifetime, suggesting reduced ATP production and consistent with ATP5f1b protection upon stress granule formation (Fig. 3*C*).

Besides promoting the assembly of stress granules, Gαq stimulation results in locomotion impacts and morphological changes in *C. elegans* neurons that may be directly traced to neurite retraction caused by calcium-dependent cytoskeletal changes, PIP_2_ hydrolysis, and endocytosis of ligand-bound Gαq coupled receptors (32, 75). Retraction of neurites will disrupt weaker synapses causing cessation of locomotion. Wolkow and coworkers have found that locomotion of *C. elegans* declines with age, and that this decline can be rescued by muscarinic agonists (6). Here, we find that the onset and increase in *C. elegans* speed is age dependent, with Day 1 worms displaying a short delay and a large increase in speed, and Day 8 worms displaying little change (Fig. 5). Egl-30 mutant worms show a lack of locomotory response to stimulation and recovery conditions showing the necessity of Gαq activity for locomotion, and important player upstream of PLCβ (21, 76). We note that Egl-30 mutant worms did not survive upon stimulation after Day 1 making it impossible to fully assess the impact of this protein. Because these age-dependent behaviors may reflect disruption of synapses upon Gαq stimulation and subsequent recovery, we characterized changes in morphology of mechanosensory neurons focusing on nerve ring disruptions and neural beading (Fig. 6). Gαq stimulation in Day 4 worms causes a small amount of nerve ring disruptions that remain fairly constant with repeated stimulation and recovery. Gαq stimulation causes a large amount of beading that is reduced with recovery and increases and recovers again with a second stimulation/recovery cycle. Day 8 worms display a large amount of nerve ring disruption even before stimulation that remains high while the large amount of beading is reduced during the first stimulation and remains high thereafter. These results suggest that beading is the main morphological change associated with Gαq stimulation and recovery. Differences between nerve ring disruption and beading may reflect contraction of neural extensions due to contraction of the surrounding muscle (Fig. 6) (77). This contraction is expected to help the recovery of retracted synapses. While middle-aged worms respond and recover quickly from these physical changes, older worms do not. These worms have impaired nerve morphology and show diverse and broad responses most likely due to their lack of Gαq response and muscle contraction. These studies highlight the robust stimulation of middle-aged worms when compared to older ones.

In summary, we show that repetitive Gαq stimulation reduces *C. elegans* lifespan through calcium-dependent and calcium-independent functions of PLCβ (EGL-8), that include stress granule accumulation and generation of abnormal neuron morphology. Additionally, we find that recovery from these effects declines with age. While it is doubtful that constitutively active Gαq could rescue these effects, it is possible that increasing Gαq and/or PLCβ levels would help disassemble stress granules and promote recovery.

## Experimental procedures

### *C. elegans* strains and maintenance

All strains were obtained from *Caenorhabditis* Genetics Center (cgc.umn.edu). Strains, JH3199 (gtbp-1, (as2055[gtbp-1::GFP])); NM4397 (jsls973[mec-7p::mRFP + unc-119(+)]); PQ530 (alg-1, (ap423[3xflag::gfp::alg-1]); DA823 (egl-30(ad805)); MT1083 (n488(egl-8)); and QW1166 (zfIs42 [rig-3p::GCaMP3::SL2::mCherry + lin-15(+)]). Standard culture methods were used to maintain *C. elegans* (78). All strains were grown on nematode growth media (NGM) agar plates seeded with OP50 *Escherichia coli* (*E. coli*). The strains were maintained at 20 °C.

### *C. eleg*ans crossing procedure

To take advantage of multiple fluorescent markers simultaneously, *C. elegans* strains were crossed using a mild heat-shock procedure. Strains of interest were placed on NGM OP50 plates as L1 larvae. Plates were then mildly heat shocked in either an incubator (30 °C) or placed upside down in water bath (30 °C) for 4 to 6 h. Heat-shocked L1 larvae plates were then stored at 20 °C until L4 progression. Once L4 stage was reached, all plates were observed for the presence of male *C. elegans*. Once males were identified for one strain, one male was placed with 5 hermaphrodites from another strain of interest. Once progeny formed, crossed *C. elegans* were confirmed using confocal microscopy taking advantage of each strain's unique fluorescent marker. This procedure was followed to cross strain JH3199 with strain NM4397 (JH3199::NM4397) and strain PQ530 with strain NM4397 (PQ530::NM43967).

### Synchronization assay

Worms were synchronized to observe age-related functions using a typical bleaching protocol (79). Gravid worms were collected off NGM OP50 plates, washed with M9 Buffer (3g KH_2_PO_4_, 6g Na_2_HPO_4_, 5g NaCl, 1 ml 1M MgSO_4_, 1 ml 1M CaCl_2_, 1 ml of 5 mg/ml cholesterol in ethanol, to 1L H_2_0, sterilized by autoclave), and bleached to release eggs. Bleaching solution was made fresh (diH_2_0, 5% NaOCl (35% final volume), and 5N NaOH (20% final volume)). Once released, eggs were spun down and washed with M9 buffer. Eggs were plated on fresh NGM OP50 plates and allowed to progress to L4 larvae. Once at an appropriate age, synchronized worms were moved to fresh OP50 plates and used for various assays.

### Application of stress conditions

Carbachol was used to stimulate the Gαq subunit to exhibit stress (80). Carbachol in powdered form was obtained from Sigma Aldrich (carbamoylcholine chloride, Catalog #C4382) and dissolved in water to a final concentration of l mM at a volume of 25 ml. 1 mM carbachol was aliquoted into 1 ml portions to be used for each experiment. The solutions were kept at −20 °C for storage. For single carbachol exposure, worms were washed with M9 buffer and placed into a 15 ml centrifuge tube. Worms were allowed to sink to the bottom and M9 buffer was removed. 1 ml of 1 mM carbachol was added to the centrifuge tube for 30 min at room temperature. Once stress was complete, carbachol was removed and worms were washed in M9 buffer and moved to a fresh OP50 plate for recovery periods at room temperature. For repeated carbachol stimulation, this process was repeated with the same *C. elegans* for two or three times with recovery periods in between. For heat shock, worms on an NGM OP50 plate were placed upside down in an oven at 35 °C for 15 min. All worms were selected at random to conduct analyses using large sample sizes.

### Locomotion assay

To access locomotion patterns, worms were collected and analyzed using WormLab and analyzed using WormLab Software (MBF Bioscience). Worms were placed on NGM dishes with or without OP50 depending on the condition. After 30s of adaptation, worms’ locomotion was recorded using a Basler acA2500-14um USB 3.0 camera with the ON Semiconductor MT9P031 CMOS sensor with a resolution of 5MP at 14 frames/s. Camera settings were adjusted to capture all worms on the plate. Wormlab is a software that enables tracking and image analysis to collect animal data from videos to enable data automation. Each plate was recorded for 1-5 min at room temperature and exported as an image sequence. To analyze, each sequence was converted to a project by setting the plate width, thresholding, and manually selecting worms for the analysis to obtain locomotion data. Each plate was analyzed for peristaltic speed in um/s, and each worm speed was recorded. Data were exported as a.csv files and further manipulated in Microsoft Excel and GraphPad Prism software.

### Lifespan assay

Worms were assessed for lifespan measurements using a manual counting assay. Strains of interest were synchronized (See Synchronization Assay) and all released eggs were allowed to grow to the L4 stage. Once at L4, a set number of worms were moved to a fresh NGM OP50 plate. After 24 h on that plate, manual counting began at Day 1 adult stage. Worms were moved over to fresh OP50 plates every day for the first week until adults reached Day 6 and no longer laid eggs to ensure the correct generation was analyzed. From Day 6, worms were then moved every other day and manually counted until lifespan was complete. Dead worms were identified with the lack of response to gentle prodding of a platinum wire near their head region. Dead worms were removed from the plate to ensure accurate count.

### Preparation for fluorescent microscopy

To image, worms were placed onto an agar pad using 5% noble agar on a cover slip with a paralytic agent for viewing. To paralyze, worms were placed in 0.5uL of 100uM tetramisole hydrochloride (Sigma Aldrich, Catalog #L9756) prepared in diH_2_0. For each 0.5uL drop, 2 to 8 worms were placed before the drop was dry. Once worms were placed on the agar pad, another cover slip was placed on top to ensure worms stayed hydrated during imaging.

### Particle analysis measurements

Worms were imaged using a 2-photon MaiTai laser (Spectra-Physics) (excitation 800 nm at 80 MHz) and a Nikon inverted confocal microscope in an ISS Alba System. Worms were imaged using a 60x water objective to microscopically count the number of particles per μm^2^ formed under different conditions. For each condition, 10 to 15 worms were prepared on agar slides (See Preparation for fluorescent microscopy) and z-stack measurements were taken at 1.0 μm per frame of the worm's head region and analyzed for particle accumulation. All z-stack measurements were combined to generate a three-dimensional image for each sample before analyzing the number of particles per sample and averaging the results.

### Number and brightness measurements

Number and brightness (N&B) measurements and theory are fully described previously (52). Images were collected using a 2-photon MaiTai laser (Spectra-Physics) (excitation 800 nm at 80 MHz) and a Nikon inverted confocal microscope in an ISS Alba System with a 60x water objective. All images were collected in the RICS and N&B element of VistaVision software. Experimentally, we collected 200 worm images at a rate of 4 μs per pixel. The images obtained for each worm were 256x256 pixels. N&B data were analyzed with SimFCS software (www.lfd.uci.edu) and regions of interest (256 × 256 box) were analyzed from a 320 × 320-pixel image where the size of the SimFCS4 boxes used was determined by the amount of monomeric protein as seen in unstimulated control worms. Any pixels on the B vs I plot that were outside of this region were determined to be protein aggregation due to the stress applied. Percent aggregation was calculated based off of total number of pixels outside of the control region, divided by the total number of pixels in the cell. The percent aggregation corresponding to either oligomerization (large aggregates) or aggregated monomeric protein (small aggregates) are also reported using text in the respective color.

### N&B analysis

N&B defines the number of photons associated with a diffusing species by analyzing the variation of the fluorescence intensity in each pixel in the cell image. In this analysis, the apparent brightness [B], in each pixel is defined as the ratio of the variance, σ, over the average fluorescence intensity <k>.

B=σ2/<k>,and<k≥εn

where n is the number of fluorophores. The determination of the variance in each pixel is obtained by rescanning the cell image ∼100 times as described earlier. The average fluorescence intensity, <k>, is directly related to the molecular brightness, ε, in units of photons per second per molecule, and n. B can also be expressed as

B=ε+1.

The apparent number of molecules, N, is defined as

N=ε/(ε+1).

### Confocal imaging

Confocal images of worms mounted on #1.5 coverslips were obtained using a Zeiss LSM510 Meta inverted confocal microscope. Worms head regions were captured using a x40 (water objective) and acquired using z-stacks with 1um thick slices to capture the whole head region. Images were analyzed using ImageJ software by combining all slices and calculating the average fluorescence intensity.

### Fluorescence lifetime imaging measurements (FLIM)

Phase modulation FLIM measurements were performed on the dual-channel confocal fast FLIM (Alba version 5, ISS Inc) using a two-photon titanium-sapphire laser and a Nikon Eclipse Ti-U inverted microscope as described (18). The lifetime of the laser was calibrated each time before experiments by measuring the lifetime of Atto 425 in water with a lifetime of 3.61 ns at 80 MHz. Worms were excited at 740 nm for intrinsic measurements, and emission spectra were collected through a 525/50 bandpass filter. For each measurement, the data were acquired using FastFLIM mode on VistaVision software.

### Immunohistochemistry

For immunohistochemistry, a procedure was adopted from previously established method (81, 82). *C. elegans* were first synchronized using the synchronization protocol (see above) and collected on Day 1, Day 4, and Day 8 with 1 ml of M9 buffer, spun down, and M9 was removed. Worm pellets were washed 3 times to remove any bacteria. 1 ml of cold boiling extraction buffer was added to the worm pellet (20 mM potassium phosphate, pH 7.4, 2 mM EDTA, 1% Triton-x-100, and protease inhibitors (ThermoFisher Scientific cat#87786)). Worms were boiled for 5 min at 95 °C and vortexed for 5 s every minute. Extraction buffer was removed, and worms were blocked in 10% fetal bovine serum in prepared antibody buffer (700 ml distilled water, 100 ml 10X PBS, 5 ml Triton X-100, 2 ml 0.5 M EDTA (pH 8.0), 1g BSA, 5 ml 10% sodium azide solution, pH 7.2 and brought to final volume of 1L with distilled water) for 1 h. Worms were probed with primary ATP5f1 (Abcam ab#117991) prepared in antibody buffer at a 1:500 dilution overnight at 4 °C. Worms were washed x3 with DPBS and spun down between washes. Worms were then probed with Alexa Fluor 488 nm goat anti-mouse (ThermoFisher Scientific cat#A11001, Lot#1572559) prepared in antibody buffer at a 1:2000 dilution for 2 h. Worms were washed x3 with DPBS and mounted on untreated microscope slides with 10ul of DPBS. Once on slide, worms were allowed to settle for 1-2 min and then covered with a 1.5 mm coverslip and frozen in dry ice. Slides were kept on dry ice for 5 min then sealed for confocal microscopy.

### Statistical analysis

Data were analyzed with GraphPad Prism 10 statistical packages, which included the student’s *t* test and one-way analysis of variance (ANOVA).

## Data availability

All data are available upon request.

## Supporting information

This article contains supporting information.

## Conflict of interest

The authors declare the following financial interests/personal relationships which may be considered as potential competing interests.
