# Supplementary material for: Repeated activation of Gαq has a detrimental impact on C. elegans in an age-dependent manner
Source: J Biol Chem. 2025 Jul 25;301(9):110518. doi: 10.1016/j.jbc.2025.110518 (PMC12926020; doi:10.1016/j.jbc.2025.110518)
Supplement: Supplementary Material [file mmc1.pdf]

Supplemental Figure 1. ***Stress granule accumulation during lifepsan studies*** Aggregation of G3BP1 in Day 1, 2 and 3 adult worms head neurons. Confocal images taken under control conditions, immediately following stimulation of Gαq , and immediately following 1 hour of recovery. Data were visualized using GraphPad Prism and analyzed using unpaired t-tests where "ns" correlates to non-significance, (***) represents $P \leq 0.001$, and (****) represents $P \leq 0.0001$. Statistical analysis is conducted by comparing treated groups to control conditions in each age worm. For all conditions, n= 8-13.

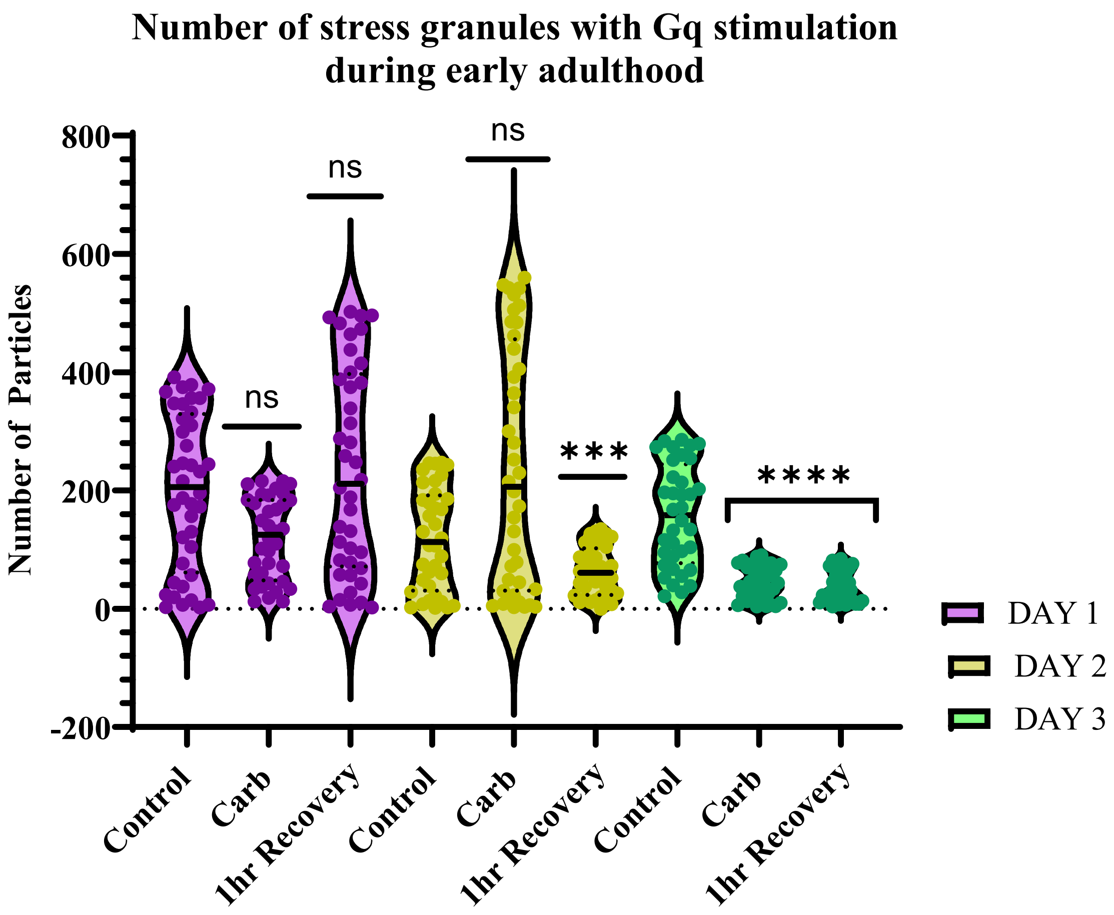

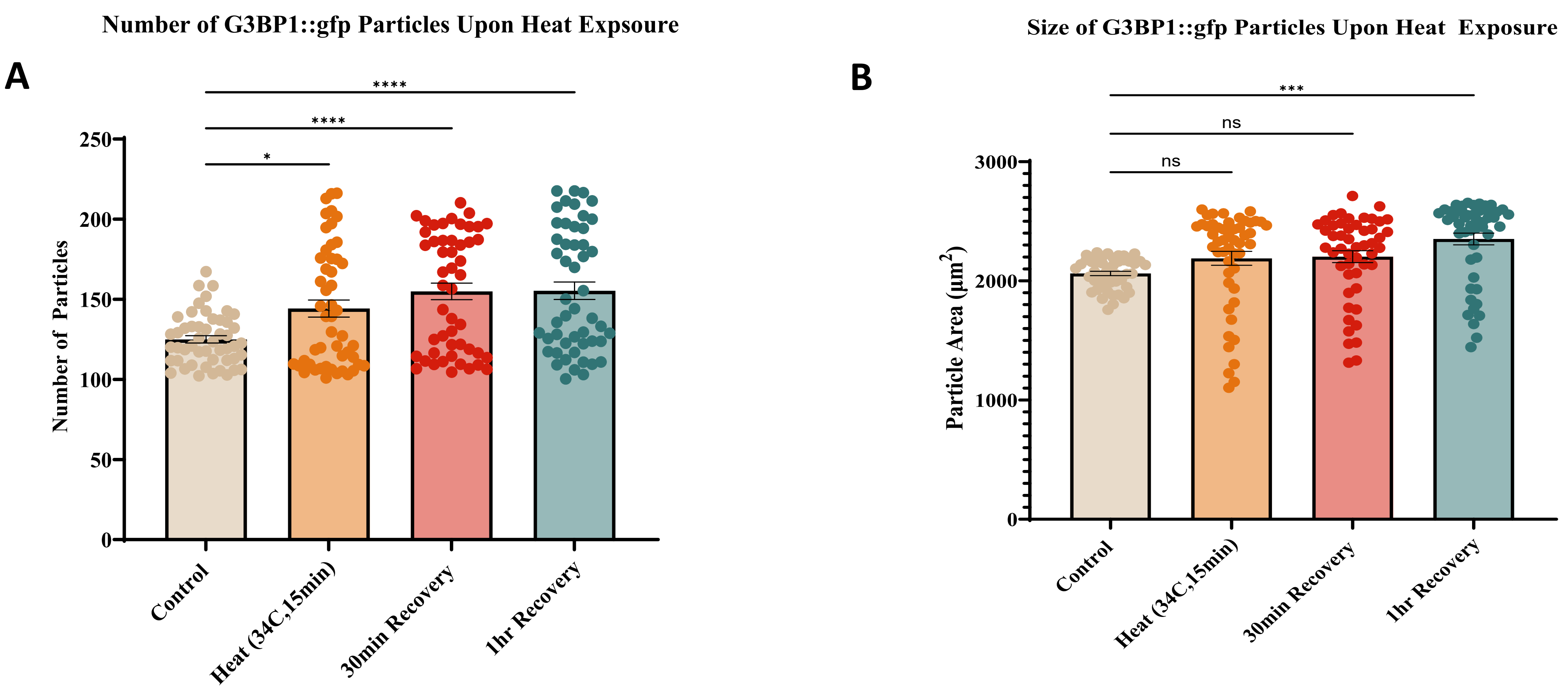

Supplemental Figure 2. *Number and size of G3BP stress granules upon heat exposure.* The number (**A**) and size (**B**) of G3BP1::gfp aggregation in *C. elegan* head neurons were compiled using confocal microscopy under control conditions, immediately following heat exposure at 34°C for 15min, and recovery. Worms were placed on NGM OP50 plates upside down in the oven with parafilm during heat stress. Worms were then removed from heat and allowed to recover for 30minutes or 1hr on the same plate at room temperature. Data were visualized using GraphPad Prism and analyzed using an ordinary One-Way Anova, where "ns" correlates to non-significance, (*) represents $P \leq 0.1$, (***) represents $P \leq 0.001$, and (****) represents $P \leq 0.0001$. For all conditions, n= 8-10 and one independent experiment was conducted (N=1). SEM values are shown.

Supplemental Table 1

Days corresponding to the % worms living after a single carbachol stimulation. Controls were unstimulated and the Days designated on the top row correspond to the day that the worms were stimulated. N values are for single dishes containing 89-103 worms. Values in red refer to significant differences.

**% Worms Alive**	**Control** ***n=5***	**Day 1** ***n=5***	**Day 3** ***n=3***	**Day 6** ***n=2***	**Day 8** ***n=2***
**70**	6.2 ± 0.4	6.8 ± 0.3	6.2 ± 0.4	6.0± 0.5	6.0 ± 0.5
**30**	10.0 ± 0.3	13.0 ± 0.4	12.7 ± 0.3	10.5 ± 0.5	10.0 ± 0.5
**10**	13 ± 0.1	16.7 ± 0.3			

	Condition	Total SG Aggregation (%)	Standard Error
**Day 1**	Control	26.43	± 4.55
	Stimulation	25.96	± 4.80
	Recovery (1hr)	35.54	± 5.91

	Condition	Total SG Aggregation (%)	Standard Error
**Day 4**	Control	41.36	± 6.14
	Stimulation	34.88	± 5.47
	Recovery (1hr)	39.85	± 3.64

	Condition	Total SG Aggregation (%)	Standard Error
**Day 8**	Control	41.70	± 6.57
	Stimulation	48.55	± 13.1
	Recovery (1hr)	34.28	± 5.87

Supplemental Table 2. *Aggregation results of number and brightness studies*. Day 1, 4, and 8 worms were treated with 1mM carbachol for 30 min to stimulate Gαq and allowed to recover on OP50 plates for 1hr. Total aggregation was determined by summing the large and small aggregation values determined by N&B analysis.
